# Cultural adaptation of the mental health first aid guidelines for depression used in English-speaking countries for China: a Delphi expert consensus study

**DOI:** 10.1186/s12888-020-02736-4

**Published:** 2020-06-26

**Authors:** Shurong Lu, Wenjing Li, Brian Oldenburg, Yan Wang, Anthony F. Jorm, Yanling He, Nicola J. Reavley

**Affiliations:** 1 Jiangsu Provincial Centre for Disease Control and Prevention, Nanjing, 210009 China; 2 Nossal Institute for Global Health, Melbourne School of Population and Global Health, University of Melbourne, Melbourne, Victoria 3000 Australia; 3 Centre for Mental Health, Melbourne School of Population and Global Health, University of Melbourne, Melbourne, Victoria 3010 Australia; 4 Shanghai Mental Health Centre, Shanghai, 200030 China

**Keywords:** Depression, Mental Health First Aid (MHFA), Cultural adaptation, Delphi study, China

## Abstract

**Background:**

Most people who meet the criteria for a diagnosis of depression in China do not receive treatment**.** Family and friends can play a role in recognising the signs of depression and encouraging the person to seek treatment. However, many of them may lack the knowledge and skills to offer such help. The aim of this study was to culturally adapt the existing English-language mental health first aid (MHFA) guidelines for helping a person with depression to the Chinese context.

**Methods:**

A Delphi expert consensus study was conducted, in which two Chinese expert panels of mental health professionals (with experience in the field of clinical management of depression, *n* = 37) and consumers and carers (with lived experience, *n* = 30) rated the importance of actions that could be taken to help a person experiencing depression in mainland China.

**Results:**

Data were collected over 3 survey rounds. In the 1st round questionnaire, 175 statements translated into Chinese from the English-language guidelines were presented to the expert panels and 12 new statements were generated from panellists’ comments. Of these 187 statements, 173 were endorsed for inclusion in the adapted guidelines for China.

**Conclusions:**

Although the adapted guidelines were still quite similar to the guidelines for English-speaking countries, they also incorporated some new actions for the Chinese context, including those relating to different ways of respecting the autonomy of a person with depression and the role of their families. Further research is needed to explore the use of these guidelines by the Chinese public, including how they may be incorporated in Mental Health First Aid training.

## Background

Depression is a common mental illness, which significantly affects people’s quality of life, health and relationships, thereby contributing to poorer functioning at work, school and within the family [1]. Worldwide, an estimated 300 million people are affected by depression every year [2]. The latest Chinese National Mental Health Survey reported the 12-month prevalence of depressive disorders among adults to be 3.6%, which may represent an increase and is likely to be due to rapid economic and social changes that have occurred in recent decades in China [3, 4].

While lack of treatment typically results in poorer clinical, social and socioeconomic outcomes, the treatment gap for mental disorders is large [5], particularly in the case of common mental disorders in lower-income countries [6]. A World Health Organisation review of 37 studies estimated that 56% of people with depression are untreated [5]. In China, people with mental disorders mostly seek help from general physicians or mental health professionals, while some may seek help from spiritual advisors [7]. Overall, low treatment rates for mental disorders have been commonly reported in metropolitan and rural areas [3, 8]. For example, a 7-year longitudinal study conducted among people aged 45 and older in 28 provinces of mainland China found that only less than 5% of those with depressive symptoms were aware of their condition and less than 2% sought professional help over the years [9], suggesting that depression remains poorly managed despite efforts by the Chinese government to improve this in recent decades [3, 10, 11].

While the reasons for the large treatment gap are still not completely understood, they can be considered in terms of structural factors (e.g., availability of services) and individual factors, including low mental health literacy, negative attitudes toward people with mental illness and low perceived need for treatment [12]. In China, low mental health literacy [13, 14] and widespread negative attitudes [15] among members of the public have been consistently identified by policymakers, researchers and healthcare professionals as major barriers to service use. These factors are also addressed in China’s recent national mental health policies. For example, *the National Work Plan for Mental Health 2015–2020* [11] calls for an increase in mental health literacy in the general population, while the first N*ational Mental Health Law* [10] formally advocates for respect for the human rights of people with mental illness.

In recent decades in high-income countries (HICs), concerns about the extent to which individual factors contribute to the treatment gap has led to the development of interventions designed to address these by improving knowledge and awareness and encouraging those affected by mental health problems to seek treatment [16] or by improving the capacity of those in an affected person’s social network to provide support [17]. There is increasing evidence that interventions targeting a person’s social network are a promising approach for promoting professional help-seeking [18], because it is common for people affected by mental illness to seek for help from their social network and also for members of the public to have contact with people affected by mental illness [19, 20]. However, members of the public often lack relevant knowledge or skills or may not feel confident in providing assistance [21].

To meet this need, the Mental Health First Aid (MHFA) training program, which focuses on training members of the public in how to assist someone who is developing a mental illness or in a mental health crisis situation (e.g., suicide), was developed in Australia in 2000 [19]. Since then, this program has spread to more than 27 other (mostly high-income, English-speaking) countries and over 2.7 million people have been trained, globally [20]. Evidence shows that the MHFA program improves mental health first aid knowledge, the ability to recognise a mental disorder, beliefs about effective treatments, confidence and intentions to provide mental health first aid, and the amount of help provided [22, 23]. MHFA training has also been found to be an effective anti-stigma intervention [22, 23]. Several studies conducted in Chinese-speaking communities in Hong Kong and Australia have shown similar effects [24, 25].

The content of the current MHFA training course is based on guidelines for how members of the public might help people with a wide range of mental health problems (e.g., psychosis, depression or trauma) [26, 27] and crises (e.g., suicide) [28]. These guidelines were developed using Delphi expert consensus studies, involving groups of experts from HICs [29]. However, Delphi studies have also been carried out to develop mental health first aid guidelines on helping a suicidal person using experts from several middle-income countries (e.g., India, Philippines and Sri Lanka) [30–32]. Comparison of these guidelines with those from HICs showed that, while there was some broad agreement across experts from different countries, there was also some cultural specificity [33]. Given differences in culture, languages and health systems between these countries and English-speaking countries, the suitability of mental health first aid guidelines developed for English-speaking countries for use in low- and middle-income countries (LMICs) is currently unknown [20]. Further tailoring of mental health first aid guidelines for LMICs is needed.

Therefore, we conducted a Delphi expert consensus study to adapt the recently-updated mental health first aid guidelines for depression used in English-speaking countries [26] for China, a country with a very different health system and cultural understanding of mental health [34, 35]. It is expected that the adaptation will provide a set of culturally-appropriate statements describing actions that Chinese community members and frontline workers can take to help a person with depression.

## Methods

### The Delphi method

We used the Delphi method to elicit consensus on potential statements to be included in the mental health first aid guidelines for depression for mainland China. The Delphi method is an iterative multistage process, designed to transform the opinions of individual experts into group consensus [36]. By using this method to develop an evidence base to guide decisions, policymakers and practitioners can move beyond relying on their own experience and draw on the accumulated experience of a larger, expert group [37]. This method has been widely used in disparate fields, including in mental health research, for example in the development of mental health first aid guidelines for English-speaking countries and some LMICs [26, 28, 30, 38].

In this study, the Delphi expert consensus survey involved four stages: (1) questionnaire development for Round 1 of the Delphi survey; (2) panel identification and recruitment; (3) data collection over 3 rounds of survey; and (4) guidelines development.

### Questionnaire development for Round 1 of the Delphi survey

The questionnaire for Round 1 was developed as follows: Firstly, the original statements endorsed from the English-language questionnaire were translated into Mandarin, with some of the translated statements modified to better reflect the Chinese context. For example, *‘to see a GP’* was replaced with *‘to see a mental health specialist’*, as in China the number of GPs is limited and they typically lack the skills to manage people with mental health problems [39]; rather, mental health specialists are responsible for providing the majority of mental health services. In addition, all phrases referring to contacting *a ‘mental health crisis team’* in the guidelines for English-speaking countries were revised to *‘contact the police’* as this is the common practice in China. The modification process involved one professional translator and four of the authors who are skilled in both English and Chinese (SL, WL, YH and WY).

There were 175 original statements included in 8 sections of the questionnaire for Round 1, which can be viewed in Table 1 of the Additional file. All statements in this questionnaire were rated for importance of inclusion in the mental health first aid guidelines for a member of the public in China to help someone who is experiencing depression.

### Panel identification and recruitment

We recruited two expert panels: one comprising mental health professionals and the other comprising consumers and carers. Mental health professionals were eligible to participate if they were psychiatrists, psychiatric nurses, psychotherapists or social workers, and had been involved in the clinical treatment/management of depression in a specialised mental health institute for at least 2 years. Evidence suggests that a panel size of 23 participants is necessary for stability of response characteristics in Delphi surveys [40]. Allowing for attrition, we therefore aimed to recruit 30 participants for each panel.

A purposive snowball sampling method was used to select participants. An initial recruitment advertisement sent out via email to personal contacts of researchers to potential professionals in the two specialised mental health institutions in Shanghai (Shanghai Mental Health Centre) and Suzhou (Suzhou Guangji Hospital). Participants were encouraged to send the email on to other eligible mental health professionals they knew.

Potential consumer and carer panellists were recruited from clinic sessions or public health lectures provided for people with affective disorders and their carers/families in the two specialised mental health institutions mentioned above. Consumers and carers were eligible to participate if they met the following criteria:

1. They had at least 1 year’s lived experience after the diagnosis of depression or 1 year’s lived experience of taking care of a person with depression on a daily basis (considering the typical course of depression and its clinical treatment used in China [41]); and
2. They had enough knowledge of (self-)management of depression, (as judged by author SL through verbal communication and clinical observation); and
3. They had at least 9 years’ school education, with adequate ability to read and write, as well as adequate understanding of how to complete the survey online.

Panellists were told that their participation was voluntary and that their responses would only be reported at the aggregate level. Panellists were reimbursed a gift card valued at RMBҰ100 for completing at least the Round 1 survey, which took 55 min on average.

### Data collection and analysis

Recruited panel members were sent a link and a Quick Response (QR) code, both of which led them to an online questionnaire hosted by Questionnaire Star (*Wen Juan Xing*, https://www.wjx.cn/) via a computer or mobile phone (by WeChat – a commonly used mobile application for social interaction in China). Participants were instructed to rate how important the helping statements were to be included in the guidelines for providing mental health first aid to a person experiencing depression. In Round 1, panellists were also encouraged to provide comments on existing statements or to suggest new helping actions that were not covered in the questionnaire.

Each statement was rated according to a five-point scale with the following options: *Essential, Important, Don’t know/It depends, Unimportant, Least important*. Statements were immediately included in the guidelines if they were endorsed by ≥80% of members in both panels as either essential or important. Statements were re-rated in the following round if they were rated as essential or important by 70–79% of either panel. Statements were immediately excluded from the guidelines if they were rated as essential or important by less than 70% of either panel.

All comments collected were sorted, translated into English and then reviewed by the working group (SL, WL, YH, NR, WY). Suggestions that contained novel ideas were used to create new statements to be included in the questionnaire of the subsequent survey round. Statements from Round 1 that met the criteria to be re-rated (i.e. being rated as essential or important by 70–79% of either panel) were also included in the Round 2 questionnaire. The Round 3 questionnaire comprised statements presented in Round 2 but requiring re-rating in a further round. Statements that still did not achieve consensus after three rounds were not included in the guidelines.

Following the first two rounds, panellists were sent a report containing a summary of the overall ratings for the statements, as well as their ratings for each statement. This allowed the panellists to compare their ratings with the level of endorsement given by the group as a whole and to inform their future ratings for those statements that needed to be re-rated.

The correlation relationship between the statement endorsement rates from the two panels was measured by Spearman’s correlation coefficient using the STATA software (version 15).

### Guidelines development

Endorsed statements (i.e. those being rated as either essential or important by ≥80% of both panels) from all three rounds were compiled. Author WL drafted the guidelines by writing the list of endorsed statements into sections of connected text. Where possible, statements were combined and repetition deleted. Statements that received comments suggesting ambiguity in the interpretation of their meaning were re-worded to make them clear and easy to understand. The draft was then circulated to members of the working group (SL, WL, YH and WY) who were native Mandarin-speakers to finalise structure and wording, creating a set of guidelines that were written in plain Mandarin and could easily be followed by members of the public in China. A number of iterations were circulated and completed before the group agreed on the final text for the guidelines.

## Results

### Expert panel information

A total of 67 expert panellists (31% male) representing the two panels of mental health professionals (*n* = 37) and consumers and carers (*n* = 30) completed Round 1 in this Delphi study. The sociodemographic characteristics of participants are shown by panel in Table 1.

**Table 1:** Characteristics of participants in Round 1 of the Delphi survey by panel

	Panel of mental health professionals	Panel of consumers and carers	All
Counts	37	30	67
Men: % (n)	41 (15)	20 (6)	31 (21)
Age (years): range (median)	27–61 (41)	20–56 (38)	20–61 (40)
University or above education: % (n)	100 (37)	97 (29)	99 (66)
Years of relevant experience: range (mean, median)	3–35 (15.8, 17)	1–37 (7.0, 4)	1–37 (11.9, 11)

The panellists were aged 20–61 years (Mean = 41, *SD* = 9; Median = 40), and all panellists except one member of the consumer and carer panel had at least a university level of education. There was a higher percentage of men in the health professional panel than in the consumer and carer panel (41% vs. 20%). Health professional panellists had an average of 15.8 years (Range 3–35, Median = 17) of experience in clinical management of depression, while the consumer and carer panellists averaged 7.0 years (Range 1–37, Median = 4) of lived experience.

Of the 37 experts in the mental health professional panel, 25 were psychiatrists, 10 psychotherapists and 2 psychiatric nurses. Most of these professional panellists also conducted research and some of them had more than one role. For example, some psychiatrists were also qualified to work as a psychotherapist in their institutions. Of the 30 members of the consumer and carer panel, 16 had lived experience as a patient with depression, 5 as a carer and another 9 had lived experience of both.

The retention rates across rounds are shown by panel in Table 2. Overall, 70% of participants in Round 1 (*n* = 47) completed Round 2 and 48% (*n* = 32) completed Round 3. The health professional panel had higher retention rates than the consumer and carer panel in both rounds 2 and 3.

**Table 2:** Participation of Delphi panellists in each round by panel

	Panel of mental health professionals	Panel of consumers and carers	All
Round 1	37	30	67
Round 2 (Retention rate over 2 rounds)	29 (78%)	18 (60%)	47 (70%)
Round 3 (Retention rate over 3 rounds)	23 (62%)	9 (30%)	32 (48%)

### Ratings of statements

**Fig. 1:**
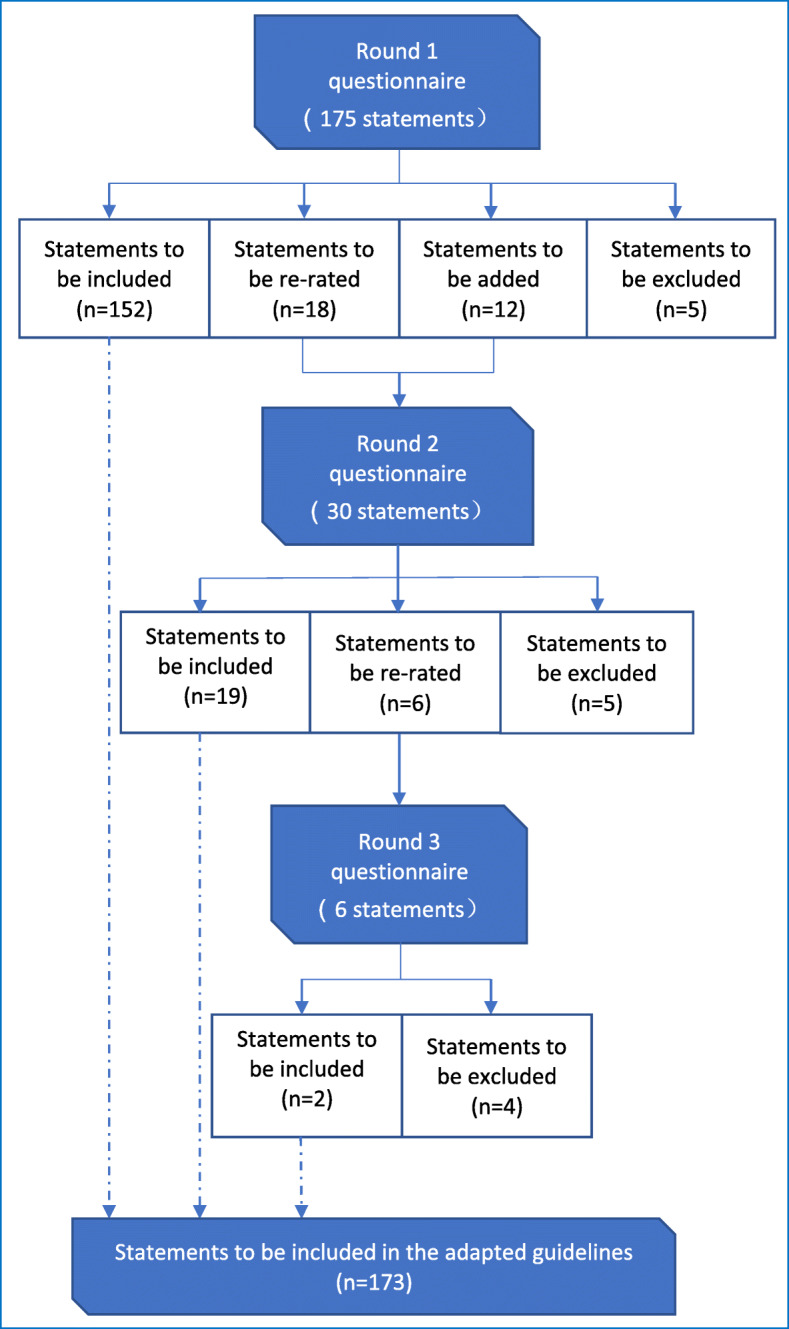
Overview of the 3 rounds of the Delphi survey

An overview of the 3 rounds of survey is provided in Fig. 1, while the full lists of statements in the questionnaires of the three rounds can be viewed in Tables 1, 2 and 3 of the Additional file. We started with 175 statements in the questionnaire in Round 1 and developed a further 12 statements from panellists’ comments, resulting in a total of 187 statements being rated across the three rounds. After 3 rounds, 14 statements from the Round 1 questionnaire were excluded (5 from Round 1 and 2, respectively, and 4 from Round 3), while all of the 12 newly-developed statements were endorsed, leading to a total of 173 statements being included in the adapted guidelines (see Table 4 of the Additional file for the full list of these statements).

The 173 statements in the adapted guidelines covered eight sections: (1) How do I know if someone is experiencing depression; (2) How should I approach someone who may be experiencing depression; (3) How can I be supportive; (4) Communicating effectively; (5) Difficulties the first aider may encounter; (6) Help-seeking; (7) What to do if the person doesn’t want help; and (8) Concerns for safety.

The consumer and carer panel had a higher endorsement rate than the professional panel (95% vs. 89%, χ^2^ = 4.856, *P* = 0.028), and the Spearman’s correlation coefficient between statement endorsement rates of the two panels was 0.53 in Round 1 (*P* < 0.001). The correlation coefficients for Round 2 and 3 were not calculated due to the unequal drop-out of the two panels.

### Differences between statements in the guidelines for China and for English-speaking countries

There were 14 statements from the guidelines for English-speaking countries that were excluded and 12 new ones that were added in the adapted guidelines. As summarised in Table 3, changes in statements happened across the eight sections of the questionnaire for Round 1. However, most changes emerged in the section ‘*How can I be supportive*’ (with 6 original statements excluded and 2 new ones endorsed), followed by the section ‘*How should I approach someone who may be experiencing depression*’ (with 4 originals excluded and 2 new ones endorsed).

**Table 3:** Changes in statements in the mental health first aid guidelines for depression for China ^a^

Changes (n)	Statements in English	Statements in Chinese
	**Section 1: How do I know if someone is experiencing Depression/Learning about depression**	**第一部分: 识别和了解抑郁症**
**Added(2)**	The first aider should consult a professional to learn more about depression.	急救人员应通过咨询专业人员来更多地了解抑郁症。
	The first aider should have some knowledge of the mental health law in China.	急救人员应了解我国精神卫生法的一些基本知识。
**Excluded(0)**	None	无
	**Section 2: How should I approach someone who may be experiencing depression**	**第二部分:怎样接近疑似抑郁者**
**Added(3)**	The first aider should not push the person too much to talk about their feelings and experiences.	急救人员不应给对方过多压力来让其谈论个人的感受和经历。
	The first aider should obtain the person’s consent before contacting their family members.	需要时, 急救人员在征得救助对象同意后联系家属或监护人。
	The first aider should think about what they want to say in advance of the conversation.	急救人员应该在对话开始前思考需要交流的内容。
**Excluded(4)**	If the first aider is worried about someone who may be depressed, they should: let the person choose when to open up.	如果急救人员担心对方可能罹患抑郁症, 则应让对方决定什么时候敞开心扉。
	The first aider should not assume that the person’s symptoms are due to depression.	急救人员不应假定对方的症状是抑郁症引起的。
	The first aider should not overwhelm the person with too much information or too many resources.	急救人员不应给对方太多信息或资源, 以防对方无法消化。
	The first aider should be open to any opportunity that presents itself to talk about their concerns with the person.	急救人员应抓住一切机会敞开心扉, 跟对方说明自己的顾虑。
	**Section 3: How can I be supportive**	**第三部分:如何提供支持**
**Added(2)**	The first aider should encourage the person to do more activities that they enjoy.	急救人员应该鼓励救助对象多从事一些他们喜欢的活动。
	The first aider should not blame themselves if the person does not get better.	即使救助对象没有好转, 急救人员也不应为此自责。
**Excluded(6)**	If the person says that they feel they are a weak person or a failure, the first aider should let the person know that: they don’t think the person is weak or a failure.	如果对方说感觉自己是个软弱的人或失败者, 急救人员应让他知道急救人员不认为他软弱或失败。
	The first aider should resist the urge to try to cure the person’s depression.	急救人员应克制想治疗对方抑郁症的冲动。
	The first aider should know that often just taking the time to talk to or be with the person lets them know that someone cares.	急救人员应明白, 只要花时间跟对方聊天或在一起, 就能让其感受到关心。
	The first aider should offer hope of a more positive future in whatever form the depressed person will accept.	无论何种形式, 只要抑郁者能接受, 急救人员应给予其憧憬未来会更好。
	The first aider should not trivialise the person’s experiences by telling them to “put a smile on their face,” to “get their act together,” or to “lighten up”.	急救人员不应告诉对方“笑一笑”、“振作起来”或“放松”以淡化他的经历。
	The first aider should not tell the person that they just need to stay busy or get out more.	急救人员不应告诉对方要保持忙碌或多出去走走。
	**Section 4: Communicating effectively**	**第四部分:有效进行沟通**
**Added(1)**	If the person does not want to talk, the first aider should consider encouraging them to write or draw.	如果救助对象不愿交谈, 急救人员可以鼓励其通过书写或画图来表达。
**Excluded(1)**	If the person finds it difficult to discuss their thoughts and feelings openly, the first aider should suggest an activity that may make it easier for them to talk, e.g. have a cup of tea, go for a walk.	如果对方很难将其想法和感受说出口, 急救人员应建议一个可以让其更容易开口的活动, 如喝杯茶、散散步。
	**Section 5: Difficulties the first aider may encounter**	**第五部分:急救人员可能会遇到的困难**
**Added(0)**	None	无
**Excluded(2)**	If the person becomes angry during the conversation, the first aider should: not make assumptions about the cause of their anger.	如果对方在交谈中变得愤怒, 急救人员应不去设想对方愤怒的起因。
	The first aider should use the following non-verbal skills to reinforce their non-judgmental communication: sit alongside the person and angled towards them, rather than directly opposite them.	急救人员应与对方坐在一起, 身体倾向他, 而不是坐在其对面。
	**Section 6: Help-seeking**	**第六部分:寻求帮助**
**Added(0)**	None	无
**Excluded(1)**	The first aider should encourage the person to make a list of questions they have to discuss with the health professional at their first appointment.	急救人员应鼓励对方在第一次就诊前, 就列出想和医务人员讨论的问题。
	**Section 7: What to do if the person doesn’t want help**	**第七部分:对方不想寻求帮助怎么办**
**Added(3)**	If the person refuses to seek or accept professional help, the first aider should tell family members about any precautions to take.	如果对方拒绝寻求或接受专业帮助, 急救人员应该告知其家属注意事项。
	If the person refuses to seek or accept professional help, the first aider should inform the person’s family or another trusted person.	如果对方拒绝寻求或接受专业帮助, 急救人员可以与其商量, 通知其信任的人或家属以获得帮助。
	If the person is not willing to seek professional help, the first aider can leave information about this with them.	如患者目前不愿就诊, 急救人员应给患者留下就诊途径, 待患者需要时可以使用。
**Excluded(0)**	None	无
	**Section 8: Concerns for safety**	**第八部分:安全方面考虑**
**Added(1)**	If the person is at risk of harming themselves or others, the FA should contact the person’s family to inform them about the risk.	如果救助对象可能会伤害自己或他人, 急救人员应联系其家属或监护人, 告知风险。
**Excluded(0)**	None	无

^a^A total number of 12 statements being added and 14 excluded across the eight sections

## Discussion

The aim of this study was to culturally adapt the mental health first aid guidelines for depression used in English-speaking countries for China. This was achieved by a 3-round Delphi survey, involving mental health professionals and consumers and carers. This study reveals similarities and differences between guidelines for China and English-speaking countries and points to important considerations for future use of the adapted guidelines.

### Comparison with the guidelines for English-speaking countries

Many similarities between the English-language guidelines and the Chinese guidelines were found. The endorsement rate of initial statements included in the Round 1 questionnaire was high (92%, 161 out of 175 statements being endorsed), suggesting a wide agreement on providing mental health first aid to people with depression between China and English-speaking countries.

Nonetheless, there were also a number of important differences, which were best reflected by exclusion of the 14 statements from the guidelines for English-speaking countries and inclusion of the 12 new statements developed specifically for the Chinese context (see Table 3). A prominent issue illustrated by these differences related to the autonomy of the person with depression. For example, experts from China proposed two new statements of *‘The first aider should not push the person too much to talk about their feelings and experiences’, ‘If the person is not willing to seek professional help, the first aider can leave information about this with them’* (i.e., respect the person’s choice not to seek for professional help immediately)*,* and both of these statements were highly endorsed in the subsequent round (endorsement rates were 89.4% and 97.7%, respectively). In contrast, some statements in the guidelines for English-speaking countries, such as *‘The first aider should be open to any opportunity that presents itself to talk about their concerns with the person’* and *‘The first aider should know that often just taking the time to talk to or be with the person lets them know that someone cares’* (i.e., to provide support without taking into account the person’s feeling), were consistently rejected by both panels. Given that mental illness is often highly stigmatised in Chinese society [15], it is often considered a very ‘private’ issue for the person (and sometimes even for their families), so that helping actions without agreement of the person could be considered humiliating and intrusive, rather than being perceived as supportive or caring. This very important cultural difference in community attitudes towards mental illness suggests that a person providing mental health first aid in China should pay significant attention to the person’s autonomy and their willingness to talk about a very personal issue such as mental illness. Also, they should give greater consideration to the issues of potential shame and stigma [42].

For a long time in China, involuntary admission and treatment for mental health problems have generally been accepted as a necessary measure to protect patients, others, and society [43], whilst the rights of people with mental illness related to admission and treatment procedures have been largely overlooked [44]. However, in recent decades, with the national reforms in the field of mental health [3, 10, 11] and rapid development of research on mental health literacy [14], respect for the autonomy of people with mental illness has attracted more attention. This Delphi study shows that the core ideas of MHFA training, such as respect, non-judgement, sympathy and understanding to people with mental health problems [19, 20], were widely endorsed by Chinese experts, with extra statements on the autonomy of the person being included. Therefore, it is proposed that the adaptation of the guidelines and their dissemination may further contribute to the issue of respecting the autonomy of people with mental illness in Chinese society.

However, it is important to keep in mind of the gap between ‘knowing’ and ‘changing’. In many LMICs, such as China, attitudes of the public towards people with mental disorders are often associated with prejudice and discrimination and they are influenced by strong traditional values related to opinions on mental illness [15]; hence, we should not assume that respecting the autonomy of the person and the core ideas of MHFA training (e.g., respect, non-judgement, sympathy and understanding) would be incorporated into Chinese first aiders’ value system or reflected in their helping actions simply by inclusion of relevant statements in the guidelines. However, it is likely that MHFA training based on the guidelines can play a role in achieving change.

A further difference between guidelines for China and English-speaking countries relates to the role of families in the process of providing mental health first aid. Panellists agreed that families should be involved and contacted if the person *‘refuses to seek or accept professional help’,* ‘*is at risk of harming themselves or others’* or simply *‘if needed*‘, although the term *‘if needed’* is ambiguous and is likely to vary from person to person. By contrast, the participants in the development of the guidelines for English-speaking countries advised the involvement of public services (e.g., GP, the police or mental health crisis teams) in similar circumstances. In Chinese society, mental illness is considered not only a personal problem but also a family issue, so it is common for families to assume primary responsibility for the care of a mentally ill member [35]. Another reason for such differences could be the lack of community mental health services and social support systems for people with mental disorders, particularly for non-psychotic conditions like depression [34]. People with mental illness have no option but to largely depend on their families, particularly in a crisis situation. Additionally, in a ‘collectivist culture’ like China’s, people tend to believe that the role of the local community as a whole and the family is more important than that of individuals; therefore, it is not surprising for Chinese panellists to agree that families should be contacted ‘if needed’ (possibly subjectively judged by the first aider), rather than letting the person make the decision. However, it is possible that the role of families in caring for a person with mental illness might be changing in line with other traditional family functions (e.g., education, physical and emotional support), due to smaller family sizes caused by China’s ‘*One Child Policy’* and the rapid urbanization process happening in Chinese society [45].

Interestingly, Chinese panellists agreed that they should ‘*not push the person too much to talk*’ and should respect the person’s choice of ‘*not willing to seek professional help*’, but they failed to reach consensus on the statement *‘If the first aider is worried about someone who may be depressed, they should let the person decide when to open up’,* even after 3 rounds of the survey. This hesitation may be related to the argument on ‘who should have the right to decide if a person with mental illness should seek help or not’, which has long been controversial in China, as in many other cultures [46]. Influenced by opinions about ‘respecting the autonomy of the person’ and ‘the traditional role of families’, Chinese panellists endorsed statements supporting both sides (e.g., *‘If the person does not incline to discuss how they are feeling, the first aider should not put pressure on them to do so’* vs. *‘If the person refuses to seek or accept professional help, the first aider should tell their family members about any precautions to take’*). Accordingly, it is possible for Chinese first aiders to be caught in a dilemma when providing mental health first aid in practice when the person refuses to seek professional help: ‘leave the person to decide’ or ‘tell their families’. This raises another important issue for MHFA training in the Chinese context.

Some emerging opinions associated with recent reforms in the field of mental health in China are also reflected in the adapted guidelines. A good example of this is the inclusion of the new statement *‘The first aider should have some knowledge of the Mental Health Law in China’* (overall endorsement rate, 94%). Also, increased availability of some novel therapies for mental health problems (e.g., art or play therapy) also underlies the following new statements *‘If the person does not want to talk, the first aider should consider encouraging them to write or draw’* and *‘The first aider should encourage the person to do more leisure activities that they enjoy’*.

### Comparison of ratings between the two panels

Overall, both the professional panel and the consumer and carer panel had high endorsement rates (89% and 95%, respectively), and the results of the correlation analysis suggest a statistically significant correlation relationship between statement endorsement rates of the two panels (Spearman’s correlation coefficient = 0.53 in Round 1, *P* < 0.001). However, the correlation coefficient observed in this study is much smaller compared to those reported in similar studies in English-speaking countries. For example, Bond et al. reported a correlation coefficient of 0.95 between panels of professionals and consumers in a Delphi study to re-develop the mental health first aid guidelines for depression [26]. Such difference is likely to be due to the truncated range, as the statements with low endorsement in the English-language questionnaire were not included in the Chinese questionnaire.

Differences in opinions between the two panels (difference in the endorsement rates > ±10%), mainly in terms of professionals’ underestimating the capacity of patients with depression, were also observed. For example, professionals did not think the person’s depression would just go away without proper treatment (all professional panellists endorsed the statement *‘The first aider should not assume the person’s depression will just go away’*) nor did they think that it was necessary to seek advice from people who have recovered from depression (more than one quarter of professional panellists rejected the statement *‘The first aider should learn more about depression by seeking advice from people who have experienced and recovered from depression’*). Instead, they expressed concern about giving too much information as this could be overwhelming for the person (81% of professional panellists endorsed the statement *‘The first aider should not overwhelm the person with too much information or too many resources’*). Consumer and carer panellists had the opposite opinion on these statements. Furthermore, the consumer and carer panel gave much higher endorsement rates to statements related to positive attitudes and respectful behaviours towards people with mental illness (e.g., *‘…the first aider should tell the person about the specific changes that they have noticed in a supportive and sensitive manner*’), as well as their key role in leading the recovery from illness (e.g., *‘The first aider should know that recovery, for the most part, must be led by the person’*).

These differences suggest significant divergence of views between mental health professionals and consumers and carers, which may be partly explained by the lack of mutual understanding, sometimes even opposing attitudes, between medical professionals and patients in Chinese society [47]. Therefore, to help the adapted guidelines to better reflect the needs of future users, it is likely to be important to include consumers and carers and value their voice equally to that of health professionals in future research, something that is not yet common in China.

### Considerations for future use of the adapted guidelines

This study aimed to harness the expertise of Chinese mental health professionals and consumers and carers to inform the actions that could be undertaken by a person providing help to someone with depression in China. The adapted guidelines will be available as a stand-alone document and also used to inform the development of a MHFA training manual and curriculum content. However, before using these guidelines to inform the public, it is important to consider the following issues: Firstly, the statements in the guidelines should be interpreted as a whole, with relevant information across sections being considered in a systematic way, rather than individually. The interpretation of the guidelines should also take the health systems and cultural understanding of mental health into consideration. Secondly, the adapted guidelines provide a new framework for mental health first aid intervention, particularly for depression - a common but inadequately addressed mental health problem in China. Lastly, with future improvements in mental health services and the public’s mental health literacy and attitudes towards people with mental illness in China, the guidelines will need to be updated.

There are many methods that may be used as part of a cultural adaptation process for behavioural health interventions [48]. The Delphi method offers a systematic way of doing this and is particularly appropriate for this study, because it parallels the process used to inform the English-language guidelines [26]. There is now a need for further exploration of how the adapted guidelines, and the associated training, might be implemented in the Chinese context, including the mental health care system, existing workforce and cultural values.

## Strengths and limitations

The key strength of this study is that it ensures the adapted guidelines not only contain up-to-date recommendations for mental health first aid for depression but also reflect the cultural context of Chinese society. Another strength relates to the involvement of a diverse range of participants. The large panel sizes (37 professionals and 30 consumers and carers vs. the minimum of 23 Delphi experts recommended [40]) also helped to achieve results that were more likely to be stable and reliable.

Despite the relatively large panel sizes, there were drop-outs across the 2nd and 3rd rounds of the study. Such drop-outs have also been reported in similar Delphi studies conducted in English-speaking countries [25, 28, 38] or LMICs [30–32]. As the 1st round of the survey took approximately 1 h (55 min on average) to complete, the time commitment required for Round 1 may have deterred panellists from participating in subsequent rounds, particularly in the consumer and carer panel (retention rate of 60% over Round 2 and 30% over Round 3). Despite these drop-out rates, the recommendation of a minimum of 23 panellists was reached for both panels for the rating of all statements in Round 1 (*n* = 175, accounting for 94% of all rated statements). The reduction in consumer and carer panel size from 18 to 9 in Round 2 means that one person’s opinion carried more weight. However, this only applied to 3% of statements.

Moreover, it is important to point out that, due to the scarcity of relevant advocacy organisations or support networks in China, consumers and carers in this study were recruited individually from clinical settings, which means they are most likely to give ratings and comments based on their own lived experience, rather than on wider knowledge of this topic, as was the case with previous studies conducted in English-speaking countries [26–28, 38].

The relatively high education level of panellists (99% with at least university level education) could be a strength of the study considering the positive association between education and mental health literacy [13, 14]. On the other hand, this could also limit the generalisability of the findings to other people in the country. Similarly, given the regional diversity of China, recruiting experts from two cities rather than across the country may also limit generalisability.

## Conclusions

Through the use of the Delphi method involving local experts who were asked to agree on a minimum set of mental health first aid actions for members of the public in China to assist a person with depression, we adapted the guidelines used in English-speaking countries for China. While there were many similarities to the guidelines for English-speaking countries, the adapted guidelines also incorporate elements of importance for China, including actions relevant to the autonomy of people with depression and the role of families in the process of providing mental health first aid.

The adapted guidelines can be used as a stand-alone product by lay people needing guidance on helping a person in their social network who is developing depression. They have the potential to contribute to public knowledge and skills for earlier detection of depression, increased help-seeking behaviours and better health outcomes for people with depression. Creating opportunities for the public to learn basic mental health first aid actions, and how to implement them when needed, is a step towards more effective early intervention and treatment of mental health problems in China.

## Supplementary information

**Additional file 1.** Statements that were presented to the panels and their ratings across 3 rounds of the survey.

## Data Availability

The data supporting our findings is attached as the Additional file, which contains all the statements that were presented to the panels and their endorsement rates.

## Abbreviations

MHFA: Mental Health First Aid
HICs: High-income countries
LMICs: Low- and middle-income countries
